# Long non-coding RNA histone deacetylase 4 antisense RNA 1 (HDAC4-AS1) inhibits HDAC4 expression in human ARPE-19 cells with hypoxic stress

**DOI:** 10.1080/21655979.2021.1933821

**Published:** 2021-05-30

**Authors:** Jie Pan, Luxin Zhao

**Affiliations:** Department of Ophthalmology, ZiBo Central Hospital, Zibo City, People’s Republic of China

**Keywords:** HDAC4-AS1, HDAC4, hypoxia, HIF-1α, retinal pigment epithelium

## Abstract

Age-related macular degeneration (AMD) is resulted from choroidal neovascularization (CNV)-mediated cicatrization and vision loss. The sustained retinal hypoxia in retinal pigment epithelium (RPE) cells was reported to contribute to CNV. However, the underlying genetic regulatory network of hypoxia response in RPE is not fully understood. In this study, human ARPE-19 RPE cells were cultured under the anoxia for 24 h and later re-oxygenated in normoxia. Then the transcriptome was investigated via high throughput sequencing. We observed that long non-coding RNA (lncRNA) histone deacetylase 4 antisense RNA 1 (HDAC4-AS1) was increased in hypoxic condition compared to normal control and decreased after re-oxygenation addition, while the change of HDAC4 expression was reduced in hypoxic condition compared to normal control and up-regulated after re-oxygenation addition in ARPE-19 cells. Furthermore, HDAC4-AS1 knockdown could suppress the transcription activity of HDAC4 only in hypoxia condition, and fluorescence in situ hybridization and pull down assay indicated that transcripts of HDAC4-AS1 could substantially bind to the promoter of HDAC4 and facilitate the recruitment of HIF-1α. Finally, we also determined the specific regions of HDAC4-AS1 that contribute to the interaction with HIF-1α and the promoter of HDAC4. Taken together, these outcomes declared that HDAC4-AS1 could inhibit HDAC4 expression through regulating HIF-1α in human ARPE-19 cells with hypoxic stress.

## Graphical abstract

**Figure:**
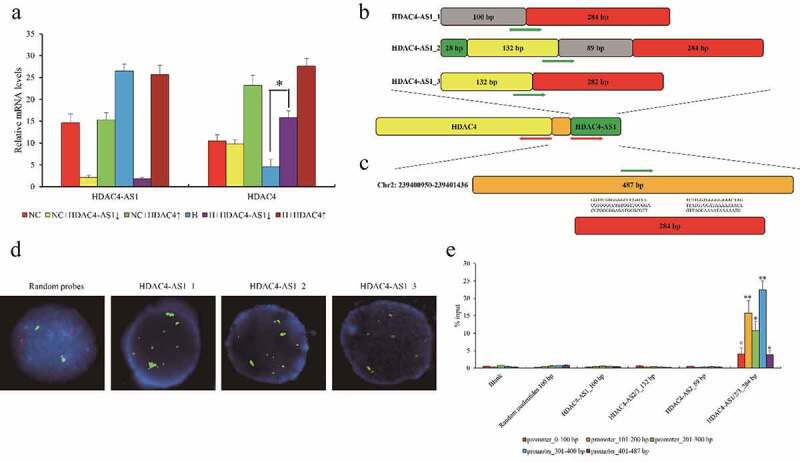

## Introduction

Age-related macular degeneration (AMD) is a common cause of blindness in older individuals. In developed countries, AMD is the leading cause of blindness in the population aged over 50 years [1]. The prevalence of AMD ranges from 2.44% to 18.98% among 45- to 89-year-old people, and the number of AMD patients had doubled in 2015 compared to 1990 [2].

Choroidal neovascularization (CNV) has been implicated in AMD, which is a kind of irreversible decrease or loss of vision caused by the degeneration of retinal pigment epithelium (RPE) cells [3]. CNV occurring in basement membrane of RPE leads to the recurrent bleeding, exudation and cicatrization in macula. Due to optical damage, metabolite deposition and nutritional deficiency, choriocapillaris perfusion usually happens in AMD, which induces a shortage of oxygen in RPE. Previous studies reported that hypoxia, as a widely recognized reason, played a crucial role in changing the blood vessel patterning of RPE cells [4,5]. Hypoxia stimulates multiple factors of revascularization, such as vascular endothelial growth factor (VEGF), angiopoietin (ANG) and nitric oxide synthase (NOS) through hypoxia-inducible factor (HIF)-mediated PI3K/Akt signaling pathway [6,7]. HIF-1 plays a crucial role in regulating gene induction to accommodate cell adaptation and survival undergoing the hypoxia challenge. HIF-1α, as a HIF family member, is stabilized under low oxygen concentrations, becomes stabilized and delivers into the nucleus to form HIF complex with HIF-1β. The activated HIF complex recognizes the hypoxia response elements in the target genes and binds to transcriptional co-activators to induce gene expression [8]. Nevertheless, the genomic binding manners of HIF-1α depend on multiple complicated factors, which are still not fully revealed [9].

Long non-coding RNAs (lncRNAs), >200 nucleotides(non-coding transcripts), play important roles in human health and diseases [10]. It had been revealed that in the AMD disease model, more than 200 lncRNAs were dy-regulated by comparing the expression profile of lncRNA in the RPE tissues of AMD patients and the control group [11]. More and more evidence indicated that lncRNAs are involved in the pathogenesis of AMD. For instance, LncRNA ZNF503-AS1 could promote RPE differentiation by downregulating ZNF503 expression [12]. Inhibition of lncRNA PWRN2 could protect multi-module-stressor-induced cell death, apoptosis and mitochondrial injuries in human ARPE-19 cells [13]. Long non-coding RNA histone deacetylase 4 antisense RNA 1 (HDAC4-AS1) is an RNA gene, and is affiliated with the lncRNA class. However, the functional role of HDAC4-AS1 in AMD pathogenesis has never been fully understood.

In this study, we cultured ARPE-19 RPE cells under hypoxic and re-oxygenated conditions, investigated the transcriptome to identify differential expressed genes, and further studied their molecular functions on hypoxic response. These findings might provide novel insight into AMD treatment.

## Materials and methods

### Cell culture

ARPE-19 RPE cells obtained from the Type Culture Collection of the Chinese Academy of Sciences (Shanghai, China) were cultured in DMEM with 10% FBS (Thermo Fisher Scientific, Waltham, MA, USA) at 37°C, 5% CO_2_ and 100% humidity. One microgram plasmids were transfected into 5 × 10^6^ ARPE-19 cells using lipofectamine 3000 (Thermo Fisher Scientific) according to the manufacturer’s instructions. After 24 h of transfection, ARPE cells were cultivated in hypoxia workstation (INVIVO2 400, Ruskinn, Bridgend, UK) with hypoxia (<0.1% O_2_, 5% CO_2_ and 95% N_2_) or normoxia (21% oxygen) for 24 h, and later re-oxygenated in an incubator with 5% CO_2_ and 95% atmospheric air. Cells were harvested after 72 h of transfection for consequent experiments.

### Nucleotides synthesis

The siRNA of HDAC4-AS1 (5ʹ-AAAGGAAUGGCCUACAAUCGA-3ʹ) and HIF-1α (5ʹ-CTACTCAGGACACAGATTTAGACTTGGAG-3ʹ) were purchased from Genepharma, and this sequence was the one with the best interference efficiency after verification. HDAC4 cDNA with EcoR I at 5ʹ and Kpn I at 3ʹ terminals were synthesized by Sangon Biotech (Shanghai, China), and cloned into PcDNA3.1 vector (Genepharma, Shanghai, China). Fluorescence in situ hybridization (FISH) probes for HDAC4-AS1 labeled with FITC (excitation wavelength 495 nm) and the promoter region labeled with R-Phycoerythrin (excitation wavelength 565 nm) were purchased from G&P Medical Enterprises (Beijing, China). Single-stranded RNA of HDAC4-AS1 with different fragments labeled by biotin was synthesized by Sangon Biotech. Primers were synthesized by Sangon Biotech. The sequences of probes, nucleotides and primers are listed in Supplementary Table 1.

### Methylthiazoletetrazolium (MTT) assay

ARPE-19 cells were sub-cultured in 96-well plates with a density of 1 × 10^4^ cells/well, treated with 0.2 mg/ml MTT (Sinopharm Chemical Reagent, Shanghai, China) for 4 h incubation, washed twice in PBS, and incubated for 10 minutes with 200 μl DMSO in dark, followed by absorbance detection at 570 nm using a microplate reader (BioTek, Winooski, VT, USA). Each concentration was conducted three individual experiments and calculated the IC50 to assess the cell viability.

### Caspase-3 activity assay

The fluorometric caspase-3 assay kit (Beyotime, Shanghai, China) was used to evaluate ARPE-19 cell apoptosis. Caspase-3 p-nitroaniline (pNA) was used to establish a standard curve. Approximately 2 × 10^6^ cells were harvested, washed with PBS, incubated with 100 μl lysis buffer on ice for 15 min with occasional vortex, and then incubated with 0.2 mM Ac-DEVD-pNA at 37 °C for 1 h. The absorbance was detected at 405 nm.

### RNA sequencing (RNA-Seq)

Total RNA of ARPE-19 cells was extracted using TRIzol (Thermo Fisher Scientific) according to the manufacturer’s protocol. An aliquot of 2 μg RNA from the cell groups under different treatment conditions were used for library preparation by using NEBNext Ultra Directional RNA Library Prep Kit (NEB, Ipswich, MA, USA) following manufacturer’s recommendations, and were then sequenced on an Illumina HiSeq platform. The raw data were adaptors-trimmed, and low-quality reads were filtered out using Trimmomatic [14]. The quality of the clean reads were checked using Fastqc [15]. Next, the clean reads were aligned to the latest human genome assembly hg38 using Hisat2 [16]. The transcripts were assembled, and the expression levels were estimated by FPKM values using the StringTie algorithm with default parameters [17]. Differential mRNA and lncRNA expression among groups was evaluated using an R package Ballgown [18], and the significance of differences was computed by the Benjamini & Hochberg (BH) p-value adjustment method. Gene annotation was described by Ensembl genome browser database (http://www.ensembl.org/index.html). The R package ClusterProfiler was used to annotate the differential genes with gene ontology (GO) terms and Kyoto Encyclopedia of Genes and Genomes (KEGG) pathways [19]. Raw data were submitted to ArrayExpress with the accession number of E-MTAB-8481.

### FISH

Slides filled with ARPE-19 cells washed by PBS were hypotension treatment in 0.075 M KCl at 37 °C for 25 min, fixed by pure ethanol for 10 min and dropped on glass slides, followed by incubating at 56 °C for 60 min. The slides were washed twice with PBS, dehydrated with 70%, 85% and 100% ethanol for 3 min in order, and naturally dried. An aliquot of 10 μl probes were added and mounted immediately. The slides were then denatured at 75 °C for 5 min and 42 °C for 16 h. After removing the coverslip, the slides were incubated in 2 × SSC for 5 min and 0.1% NP-40/2 × SSC for another 2 min at 46 °C, followed by incubating in 70% ethanol at room temperature for 3 min and dried in dark. An aliquot of 15 μl DAPI was dropped and mounted again. After 10 min of incubation in dark, the slides were observed under a fluorescence microscope with appropriate filters.

### Pull down assay

In brief, 1 × 10^7^ ARPE-19 cells were treated by lysis buffer with 1% NP40 on ice for 30 min, and the supernatant was harvested by centrifugation at the highest speed for 10 min, followed by ultra-sonication with 40 cycles. Then, 50 nM Biotin-labeled RNA fragments were incubated with the supernatant at 37 °C for 30 min. Streptavidin beads were added at 4 °C for additional incubation of 30 min to capture biotin. Beads were centrifuged at 800 g for 5 min and washed by 300 mM DEPC-prepared NaCl for three times. The eluted DNA was used as the template to detect promoter region by quantitative PCR (qPCR), and the interacted HIF-1α was detected by western blot.

### Chromatin immunoprecipitation (ChIP) assay

Briefly, 5 × 10^6^ cells were fixed with 1% formaldehyde, quenched with 0.125 M glycine at room temperature, and then lysed in 500 μl lysis buffer (10 mM Tris-HCl (pH 8.0), 10 mM NaCl, and 0.2% IGEPAL CA-630 (Thermo Fisher Scientific)) on ice for 30 min. The genomic DNA was sonicated into 200–500 bp. Ten percent of each whole-cell lysate was stored as input, and the rest of the lysate was incubated with 1 μg of the primary antibodies of HIF-1α (Cat. No. 36169, Cell Signaling Technology, Beverly, MA, USA) at 4°C overnight. An additional 2-hour pull down was performed at 4°C with protein-A beads (Thermo Fisher Scientific). After washing by 600 mM LiCl, centrifuging at 800 g for 5 min and discarding the supernatant for three times, the purified DNA was used to perform qPCR to detect the enrichment of HIF-1α.

### RNA immunoprecipitation (RIP) assay

RIP was performed using a RNA-Binding Protein Immunoprecipitation Kit (Millipore, Cambridge, MA, USA) according to the manufacturers’ protocol. The harvest cells were lysed in immunoprecipitation lysis buffer, and anti-bodies against HIF-1α and IgG (EMD Millipore) were added and cultured with the cell extract overnight under 4°C. Then, streptavidin-coated magnetic beads were added for incubation for 2 h. The isolated and purified RNAs in which HDAC4-AS1 might be enriched using qPCR analysis.

### qPCR assay

The cDNA templates taken from RNA reverse transcription were detected the target gene transcription using FastStart Universal SYBR Green Master (Roche, Basel, Switzerland). In accordance with the given instructions, 95°C for 30 s for initial denaturation, followed by 40 cycles at 95°C for 5 s, appropriate annealing temperatures of 10 s and 72°C, then 30 s were set up for PCR conditions. Ct values were harvested and calculated by using the delta-delta method. The expression of GAPDH was used as a quality control.

### Western blot

ARPE-19 cells were harvested and placed in cold radioimmunoprecipitation assay buffer (Beyotime) containing freshly prepared 2 mM PMSF and 1 X protease inhibitor cocktail (Beyotime). Aliquots of proteins (40 µg) were loaded into the lanes of a SDS polyacrylamide gel, and the proteins separated through electrophoresis were transferred onto nitrocellulose membranes. Subsequently, the membranes were blocked with 5% nonfat dry milk in 0.01 M PBS buffer (pH 7.4) and 0.05% Tween-20 for 1 h at room temperature. Afterward, the blocked membranes were incubated with appropriate primary antibodies against HIF-1α (1:2000), VEGFA (1:2000, Cat. No. ab46154, Abcam, Cambridge, MA, USA), and ANG1 (1:5000, Cat. No. ab95230, Abcam) overnight at 4 °C, and then with anti-rabbit or anti-mouse secondary horseradish peroxidase-conjugated antibodies (Amersham, Arlington Heights, OH, USA). GAPDH (1:5000, AF5001, Beyotime) was used as the loading control. The expression was determined using the enhanced chemiluminescence method (Amersham), and the density of immunoblots was measured with Quantity One software (Bio-Rad Laboratories, Hercules, CA, USA).

### Statistical analysis

All statistical analyses were performed in SPSS 20. Delta-delta method was used to normalize the real-time PCR data. One-way ANOVA was used to analyze the difference among groups. The *p-*value less than 0.05 was considered as statistical significance.

## Results

### RNA profiling of RPE cells responding to oxygen

**Figure 1.**
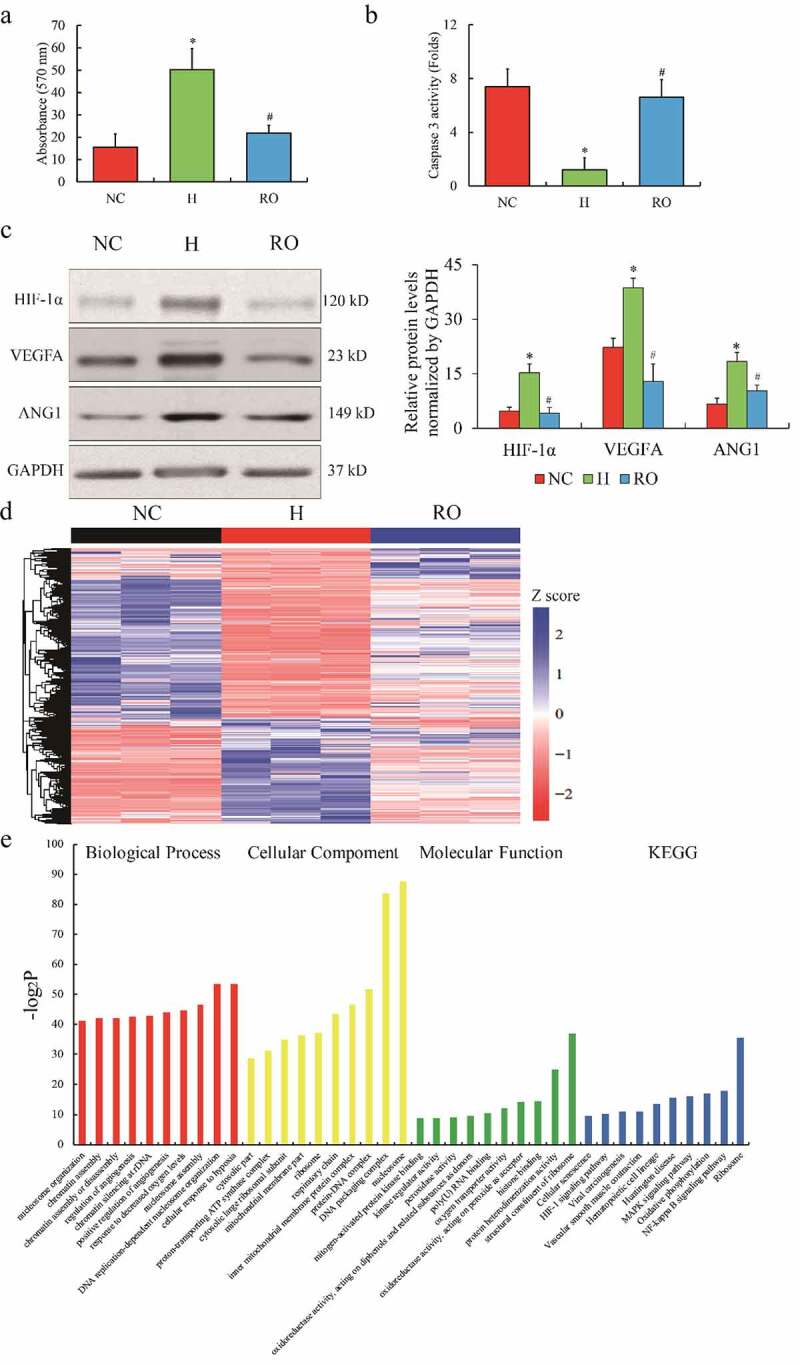
The RNA profiles of ARPE-19 cells treated by hypoxia. The phenotype of cell growth (a) and apoptosis (b) of cultured ARPE-19 cells under hypoxic and re-oxygenated conditions. The protein expression of HIF-1α, VEGFA and ANG1 in ARPE-19 cells (c). Heatmap of differential expressed genes of ARPE-19 cells (d). Color bars above the heatmap represent sample groups: red is for up-regulated genes and blue is for down-regulated genes. Gene ontology analysis including biological process, cellular component and molecular function and KEGG analysis (e) of the top 10 function enrichments or pathways associated with these differential expressed genes. NC means normoxic condition, H means hypoxic condition and RO means re-oxygenation. The comparison of H to NC (‘*’) and RO to H (‘#’) with the statistical significance that *p* value is less than 0.05. Data are presented as mean ± standard error of the mean of three individual experiments

Initially, ARPE-19 cells were treated with hypoxic stress (1% O_2_) and re-oxygenation (21% O_2_). The features of the hypoxia challenge and the re-oxygenation effect on ARPE-19 cells were identified. The proliferation ability of ARPE cells was enhanced by hypoxia (H) compared to normoxic control (NC), while compromised by re-oxygenation (RO) (Figure 1a); cell apoptosis displayed an opposite phenotype under hypoxia and re-oxygenation (Figure 1b). HIF-1α, VEGFA and angiopoietin-1 (ANG1) were observed highly expressed in H group, but down-regulated in RO group (Figure 1c). Next, the RNA profiling of NC, H, and RO groups was characterized. Deep sequencing of mRNA libraries generated a total of 245.2 M reads of NC, 245.6 M reads of H, and 245.6 M reads of RO. Approximately 92.46% of the reads were uniquely mapped to the human genome 38 (Ensemble Genomes release 92), which indicated a qualified library preparation without poly-A selection (Supplementary Table 2). The gene expression with FPKM distribution (Figure 1d) presented the differentially expressed genes among the three groups. A total of 1848 mRNAs and 345 lncRNAs were up-regulated (fold change (FC) > 2, *p*< 0.05), while 1925 mRNAs and 541 lncRNAs were down-regulated (FC < 0.5, *p*< 0.05) in H group compared to NC group; 1054 mRNAs and 351 lncRNAs were up-regulated (FC > 2, *p*< 0.05), while 714 mRNAs and 227 lncRNAs were down-regulated (FC < 0.5, *p*< 0.05) in RO group compared to H group. Furthermore, the associated function enrichments and signaling pathways involved in differentially expressed genes include angiogenesis, oxygen metabolism and mitochondrial, as well as HIF-1α, MAPK and NF-κB signaling pathways (Figure 1e). Our observation indicated that a lncRNA named histone deacetylase 4 antisense RNA 1 (HDAC4-AS1) was highly expressed in H group compared to NC group (FC = 3.19, *p*= 1.12E-4), but down-regulated in RO group compared to H group (FC = 0.47, *p*= 0.0035). Interestingly, the change of HDAC4 (FC = 0.5, *p = *6.25E-11 in H *vs*. NC; FC = 1.45, *p = *3.69E-5 in RO *vs*. H) was the opposite to HDAC4-AS1, which indicated that HDAC4-AS1 might play a suppressive effect on HDAC4 transcription. Taken together, the findings indicated that HDAC4-AS1 might be one of the novel candidates responding to oxygen concentration in ARPE-19 cells.

### The effects of HDAC4-AS1 on HDAC4 transcription under hypoxic stress

**Figure 2.**
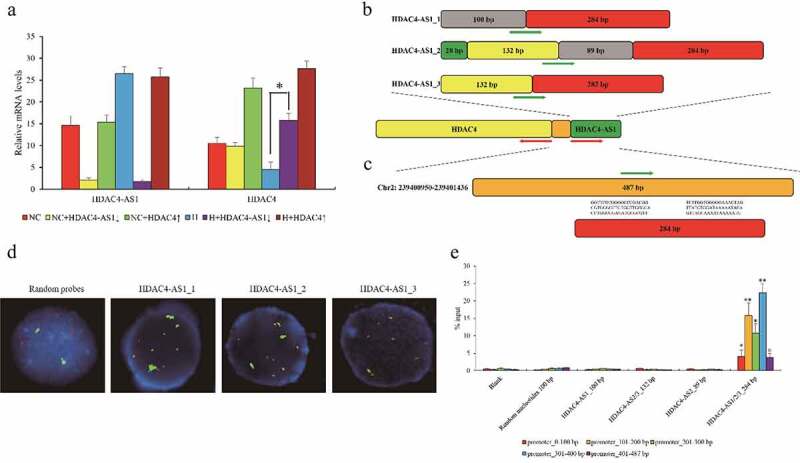
The interaction between HDAC4-AS1 and the promoter of HDAC4 in ARPE-19 cells. The expression of HDAC4 affected by silenced HDAC4-AS1 in ARPE-19 cells (a). The schematic diagram of different variants of HDAC4-AS1 (b). The putative binding sequence of HDAC4-AS1 to the shared promoter region of HDAC4/HDAC4-AS1 (c). Red arrows represent the transcription orientation. Green arrows represent the probes’ location. The interaction between HDAC4-AS1 and HDAC4 in hypoxic ARPE-19 cells shown by FISH assay (d). Green signals mean the transcripts of HDAC4-AS1, red signals mean the allelic target promoter region, and yellow signals mean the overlapped green and red signals. The interaction between HDAC4-AS1 and HDAC4 in hypoxic ARPE-19 cells shown by pull down assay (e). Data are presented as mean ± standard error of the mean of three individual experiments. NC means normoxic ARPE-19 cells, H means ARPE-19 cells cultured under only hypoxic condition, HDAC4-AS1↓ means HDAC4-AS1 knockdown, and HDAC4↑ means HDAC4 over-expression. ‘*’ and ‘**’ represent the statistical significance as *p* value is less than 0.05 or 0.01

Since highly expressed HDAC4-AS1 and down-regulated HDAC4 were observed in hypoxic ARPE-19 cells, in order to study the connection between HDAC4-AS1 and HDAC4 in ARPE-19 cells, HDAC4-AS1 was knocked down or HDAC4 was over-expressed in ARPE-19 cells under NC or H conditions. We found that HDAC4-AS1 knockdown could enhance HDAC4 transcription, while HDAC4 over-expression did not influence HDAC4-AS1 under H condition (Figure 2a). Unexpectedly, neither HDAC4-AS1 knockdown nor HDAC4 over-expression changed the expression of each other under NC condition (Figure 2a). Here, we drew two hypotheses: First, HDAC4-AS1 was the upstream regulatory factor for HDAC4 transcription activity; second, hypoxia was essential for regulating HDAC4 expression.

We noticed that HDAC4-AS1 had three different variants and it shared the same promoter region with HDAC4 from the opposite orientation (Figure 2b). Given the predicted interaction between HDAC4-AS1 and the shared promoter with high likelihood (Figure 2c) using LongTarget database [20], we designed the probes for three variants of HDAC4-AS1 and promoter region of HDAC4/HDAC4-AS1, and performed FISH in ARPE-19 cells. Remarkably, three HDAC4-AS1 transcripts could substantially interact with HDAC4 promoter, compared to the random-sequence probes (Figure 2d). Moreover, pull down assay also supported the findings from FISH, and further demonstrated that the homologous sequence (284 bp) among HDAC4-AS1 variants contributed to the interaction with HDAC4 promoter (Figure 2e). Taken together, our results revealed that HDAC4-AS1 could bind to promoter region and suppress HDAC4 transcription in hypoxic ARPE-19 cells.

### The role of HIF-1α in HDAC4 transcription regulated by HDAC4-AS1

**Figure 3.**
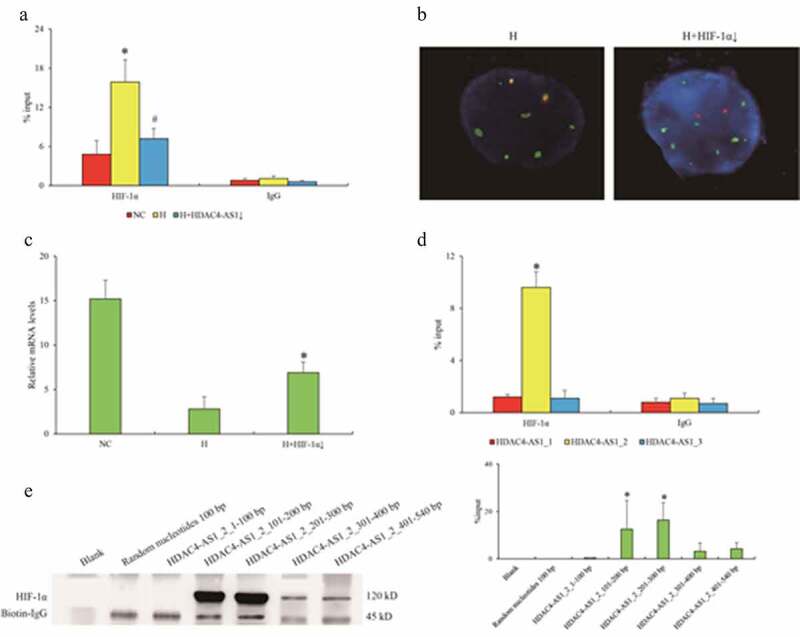
The interaction between HDAC4-AS1 and HIF-1α in ARPE-19 cells. Enrichment of HIF-1α on shared promoter of HDAC4/HDAC4-AS1 shown by ChIP-qPCR assay (a). The statistical significance by the comparison between H and NC (‘*’) as well as between H+ HDAC4-AS1 knockdown and H (‘#’), with *p* value less than 0.05. The interaction between HDAC4-AS1 and promoter region affected by HIF-1α knockdown shown by FISH assay (b). The HDAC4 transcription affected by HIF-1α knockdown (c). ‘*’ represents the statistical significance by the comparison between H + HIF-1α knockdown and H, with *p* value less than 0.05. The interaction between HIF-1α and different variants of HDAC4-AS1 (d). ‘*’ represents the statistical significance comparing HIF-1α with IgG in HDAC4-AS1_2 group. The detailed binding domain of HDAC4-AS1_2 interacted with HIF-1α shown by pull down and western blot assays (e). Data are presented as mean ± standard error of the mean of three individual experiments

To investigate the second hypothesis, we focused on HIF-1α and studied the regulatory role of hypoxia in HDAC4 transcription. ChIP-qPCR assay showed that the enrichment of HIF-1α on HDAC4/HDAC4-AS1 promoter region was significantly enhanced in ARPE-19 cells under H condition compared to NC condition, but HDAC4-AS1 knockdown could weaken the interaction with the promoter of HDAC4/HDAC4-AS1 under H condition (Figure 3a). On the other hand, HIF-1α knockdown could compromise the interaction of HDAC4-AS1 with promoter region (Figure 3b), and attenuate the transcription of HDAC4 under H condition (Figure 3c), indicating that HIF-1α might play a crucial role in suppressive regulation of HDAC4 modulated by HDAC4-AS1.

Next, we harvested the nucleus of ARPE-19 cells to exclude the inactivated HIF-1α in cytoplasm, and further performed pull down and RIP-qPCR assays to examine the interaction between HDAC4-AS1 and HIF-1α. RIP-qPCR assay verified the substantial interaction between HDAC4-AS1 and HIF-1α, while only the longest variant of HDAC4-AS1 (HDAC4-AS1_2, 540 bp) was verified to bind with HIF-1α (Figure 3d). Consistently, pull down assay showed that the key domain (101–300 bp) of HDAC4-AS1_2 contributed to HIF-1α interaction (Figure 3e). We considered that HDAC4-AS1_2 had the same sequence with HDAC4-AS1_3, except for the different domains of 28 bp at 5ʹ terminal and 89 bp in the middle (Figure 2b), while HDAC4-AS1_3 failed to bind with HIF-1α. Therefore, we speculated that the fragments of HDAC4-AS1 (161–249 bp) might facilitate the recruitment of HIF-1α onto promoter of HDAC4.

Overall, our study suggested that HDAC4-AS1 recruited HIF-1α to suppress HDAC4 transcription activity in hypoxic ARPE-19 cells.

## Discussion

The appropriate cellular hypoxia response during retinal development is important for ocular circulatory transition from embryo to adult [21]. However, in multiple retinal diseases such as AMD and diabetic retinopathy (DR), sustained ischemia and hypoxia in RPE cells eventually cause cell senescence and apoptosis. In completely developed tissues, unlike other HIFs, HIF-1α is usually absent in normoxic state because HIF-1α is marked for degradation by prolyl hydroxylase domain (PHD) and factor inhibiting HIF (FIH) proteins. When oxygen tension is reduced, PHD and FIH are inactivated, allowing for the accumulation of HIF-1α. The activated HIF-1α dimerizes with HIF-1β, forming an active HIF-1 complex and transcriptionally regulating the expression of a plenty of genes by binding to hypoxia response elements (HREs) in their promoters. Previous studies reported that lncRNA-p21 could directly bind to HIF-1α and von Hippel–Lindau (VHL) protein and facilitate HIF-1α ubiquitination, indicating that hypoxia-responsive lncRNA might play an important role in regulating gene expression via RNA-protein interaction [22].

In this study, we mainly focus on the differential expressed noncoding genes, which also contribute to the functions of proteins, the activities of signaling pathways and cell morphology and fate. We demonstrate that lncRNA HDAC4-AS1 is a regulatory target responding to oxygen concentration in RPE cells. Previous studies suggested that several types of lncRNAs could be enriched in promoters of nearby genes [23,24]. Histone deacetylases (HDACs) play important roles in CNV [25,26]. HDAC inhibition can protect the retina from acute injury, attenuate CNV and may have an inhibitory effect on CNV development [26]. HDAC4 overexpression in a mouse model of retinal degeneration prolonged photoreceptor survival, which was attributed to the activity of HDAC4 in the cytoplasm and at least partly relied on the activity of HIF-1α [27]. Previous study has also reported that HDAC4 can suppress HIF-1α acetylation and enhance HIF-1α transcriptional activity and stability in responding to hypoxia [28,29]. HDAC4 can enhance the transactivation of HIF-1a, instead of its transcription or protein translation. Interestingly, our data confirm an opposite process that HIF-1α can down-regulate HDAC4 transcription via HDAC4-AS1, indicating a putative negative feedback model of HDAC4 and HIF-1α for the epigenetic homeostasis under hypoxic stress. Expectedly, we observe that HDAC4-AS1 shares the same promoter region with HDAC4 by a different orientation, and the FISH assay demonstrates that the transcript of HDAC4-AS1 substantially binds to the promoter region (Figure 2d), and the common sequence of different HDAC4-AS1 transcripts contributes to such an interaction (Figure 2e). Moreover, we further reveal that HDAC4-AS1_2 can interact with HIF-1α (Figure 3b–e). From an evolutionary perspective, HDAC4-AS1, as a unique factor for regulating HDAC4 transcription, may be developed from an enhancer element. HDAC4-AS1 acts as a bridge between HIF-1α and HDAC4 transcriptional activity. The different transcripts of HDAC4-AS1 may modulate HDAC4 transcription responding to external stimulus via recruiting different protein factors. In this system, we indicate that HDAC4-AS1_2 has a specific region to interact with HIF-1α (Figure 3e), but the effects of the other two HDAC4-AS1s are still unknown. We speculate that the transcripts of HDAC4-AS1 can be applied to the basic regulatory model of HDAC4. Future study on proteomics at the promoter of HDAC4 may help to reveal the potential transcription factor interacting with HDAC4-AS1_1 and HDAC4-AS1_3.

## Conclusion

Our study shows the model of genetic regulation via lncRNA/DNA/protein interaction in the nucleus. We reveal a regulatory axis of HDAC4-AS1/HIF-1α/HDAC4 in RPE cells under hypoxic stress, and provide therapeutic targets for treating retinal diseases.

## Supplementary Material

Supplemental Material

## Data Availability

The datasets used and/or analyzed during the current study are available from the corresponding author upon reasonable request.
