# Supplementary material for: Long non-coding RNA histone deacetylase 4 antisense RNA 1 (HDAC4-AS1) inhibits HDAC4 expression in human ARPE-19 cells with hypoxic stress
Source: Bioengineered. 2021 May 30;12(1):2228–37. doi: 10.1080/21655979.2021.1933821 (PMC8806694; doi:10.1080/21655979.2021.1933821)
Supplement: Supplemental Material [file KBIE_A_1933821_SM2119.zip › Supplementary/Supplementary table 1.docx]

| Symbol names | Sequences |
| --- | --- |
| HDAC4 for qPCR | F: 5’-ACCCTTCCCGCCCTCCAGCAGAGGCTC-3’ |
|  | R: 5’-GCCGACTGGGTCCGCCCCAGTGGGCGGT -3’ |
| β-Actin for qPCR | F: 5’-CTCCATCCTGGCCTCGCTGT-3’ |
|  | R: 5’-GCTGTCACCTTCACCGTTCC-3’ |
| HDAC4 promoter for qPCR | F: 5’-GCCTAGCGGTCAGCACCGCATGTCTGCGA-3’ |
|  | F: 5’-CCTCACGGCCGCGTTCCCGCCGCG-3’ |
| HDAC4-AS1 for qPCR | F: 5’-CACTGTCCCCGGAATCCCGCGTTCC-3’ |
|  | R: 5’-AATGGCCTACAATCGAAGCGAACG-3’ |
| HDAC4-AS1_1 for qPCR | F: 5’-GCGCAGGGACCGGGGGCCAGTA-3’ |
|  | R: 5’-CTCCTAAAAGGAATGGCCTACAAT-3’ |
| HDAC4-AS1_2 for qPCR | F: 5’-TCCGGGTCCAGGCAGGCGTGGA-3’ |
|  | R: 5’-CTCCTAAAAGGAATGGCCTACAAT-3’ |
| HDAC4-AS1_3 for qPCR | F: 5’-TGTAGGTCCGCCAGGCCTGCAACGGC-3’ |
|  | R: 5’-CTCCTAAAAGGAATGGCCTACAAT-3’ |
| FISH probes for HDAC4-AS1_1 | F: 5’-ACGGGCCCCGGCCGTGTCCGGTCGCACTC  GGTAGCCCGCATGGCTGCCTAGCGGAGTATGAC  AACTTTTCAACTTGAGTGCACAC-3’ |
| FISH probes for HDAC4-AS1_2 | F: 5’-TCGCCGCGTCCCTGCTCAGGCGGAGGGAG  CCCGGCCCGCCGCCAGGGTCTCGAGTCCGGGTCCAGGCAGGCGTGGAGGTGGCGGGAACGGCGCACCGCCC-3’ |
| FISH probes for HDAC4-AS1_3 | F: 5’-GCCCGGCCCGCCGCCAGGGTCTCGAGT  CCGGGTCCAGGAGTATGACAACTTTTCAACT  TGAGTGCACACA-3’ |
| FISH probes for HDAC4 promoter | F: 5’-GGCGATAGGCCACCCCGCACGCCCGTCA  GCCGGCCCGGCCCCGCCCAGCCAGCCGGC-3’ |

Supplementary table 1. All primers used in this study are listed.
