# Supplementary material for: Long non-coding RNA histone deacetylase 4 antisense RNA 1 (HDAC4-AS1) inhibits HDAC4 expression in human ARPE-19 cells with hypoxic stress
Source: Bioengineered. 2021 May 30;12(1):2228–37. doi: 10.1080/21655979.2021.1933821 (PMC8806694; doi:10.1080/21655979.2021.1933821)
Supplement: Supplemental Material [file KBIE_A_1933821_SM2119.zip › Supplementary/Supplementary Table 2.docx]

| Sample names | NC_1 | NC_2 | NC_3 | H_1 | H_2 | H_3 | HC_1 | HC_2 | HC_3 |
| --- | --- | --- | --- | --- | --- | --- | --- | --- | --- |
| Raw reads | 83875968 | 80033574 | 81304518 | 80828886 | 86115750 | 78667316 | 80445630 | 83713750 | 81833090 |
| Total raw bases | 12581395200 | 12005036100 | 12195677700 | 12124332900 | 12917362500 | 11800097400 | 12066844500 | 12557062500 | 12274963500 |
| Clean reads | 80372970 | 78069880 | 78572028 | 78748646 | 83698538 | 74394836 | 76484140 | 77515480 | 74187794 |
| Total clean bases | 11667151662 | 11490579125 | 11139062150 | 11547311330 | 12306204139 | 10765269166 | 11067893435 | 11104072377 | 10337091365 |
| Mapped reads | 77319417 | 75077502 | 75238078 | 74660726 | 79356207 | 70600345 | 73237125 | 74268612 | 71116770 |
| Mapped ratio | 96.20% | 96.17% | 95.76% | 94.81% | 94.81% | 94.90% | 95.75% | 95.81% | 95.86% |
| Uniquely mapped reads | 75611523 | 73433467 | 73573636 | 71860997 | 76179274 | 67900396 | 71251333 | 72263399 | 69157619 |
| Uniquely mapped ratio | 94.08% | 94.06% | 93.64% | 91.25% | 91.02% | 91.27% | 93.16% | 93.22% | 93.22% |

**Supplementary table 2. Summary of the results of RNA-seq data in this study.** NC, H and HC represent negative control, hypoxia and hypoxia with curcumin groups. Each group has three duplications.
